# Autoimmune encephalitis after BBIBP-CorV (Sinopharm) COVID-19 vaccination: a case report

**DOI:** 10.1186/s12883-022-02949-y

**Published:** 2022-11-14

**Authors:** Miguel A. Vences, Mary M. Araujo-Chumacero, Edu Cardenas, Diego Canales, Arturo Alvarez, Ebelin Barja, Maria Fe Albujar, Diego Urrunaga-Pastor

**Affiliations:** 1 Departamento de Neurología, Hospital Nacional Edgardo Rebagliati Martins, Avenida Edgardo Rebagliati 490, Jesús María, 15072 Lima, Peru; 2 Vicerrectorado de Investigación. Universidad San Ignacio de Loyola, Unidad para la Generación y Síntesis de Evidencias en Salud, Campus 2, avenida La Fontana 750, La Molina, Lima, Peru; 3 Instituto de Evaluación de Tecnologías en Salud e Investigación–IETSI EsSalud, Lima, Peru

**Keywords:** Encephalitis, COVID-19, Vaccination, Drug-related side effects and adverse reactions (source: MeSH NLM)

## Abstract

**Background:**

Vaccination is an important public health strategy; however, many neurological adverse effects are associated with COVID-19 vaccination, being encephalitis a rare manifestation.

**Case presentation:**

We present the case of a 33-year-old woman who received the first dose of the BBIBP-CorV vaccine against COVID-19 on April 4 and the second dose on April 28, 2021. Three days after receiving the second dose, she experienced a subacute episode of headache, fever, insomnia, and transient episodes of environment disconnection. We obtained negative results for infectious, systemic, and oncological causes. Brain magnetic resonance imaging showed lesions in the bilateral caudate nucleus and nonspecific demyelinating lesions at the supratentorial and infratentorial compartments. The results of the neuronal autoantibodies panel were negative. She had an adequate response to immunoglobulin and methylprednisolone; however, she experienced an early clinical relapse and received a new cycle of immunosuppressive treatment followed by a satisfactory clinical evolution.

**Conclusions:**

We report the first case of severe encephalitis associated with BBIBP-CorV (Sinopharm) vaccination in Latin America. The patient had atypical imaging patterns, with early clinical relapse and a favorable response to corticosteroid therapy.

## Background

As of January 27, 2022, more than 356 million cases and 5.6 million deaths have been reported worldwide due to COVID-19 [1]. The lack of a specific treatment regimen has led to the development of different types of vaccines, some of which have already been approved by many countries such as the United States and the European Union [2]. On July 6, 2022, the World Health Organization (WHO) reported approximately 61.5 per 100 people worldwide are already vaccinated with two doses, generating a significant reduction in severe cases and deaths [1].

BBIBP-CorV (developed by Sinopharm) is an inactivated virus vaccine whose phase 3 clinical trials were carried out in several countries, including Peru [3]. The WHO has supported the efficacy of this vaccine and has been distributed worldwide for immunization. Neurological adverse effects, such as encephalitis, myelitis, and Guillain Barré syndrome have been reported with the different vaccines against COVID-19. Therefore, the prevalence of postvaccination adverse effects is under exploration as we write [4, 5].

The mechanism by which vaccines cause these complications has not been established yet; however, compromised humoral immunity, molecular mimicry, and aberrant immune reactions are possible hypotheses. Previous case reports have described severe postvaccination adverse effects after different COVID-19 vaccines [4–7]. However, few studies describe these adverse effects after the application of the BBIBP-CorV vaccine. Therefore, we present the case of a patient with clinical manifestations of autoimmune encephalitis after the administration of a second dose of the BBIBP-CorV vaccine (Sinopharm).

## Case presentation

We present the case of a self-sufficient 33-year-old woman, with no relevant history. She was diagnosed with COVID-19 using an antigenic test for SARS-CoV-2 in July 2020 and presented with mild symptoms without requiring oxygen or hospitalization. She received the first dose of the BBIBP-CorV COVID-19 vaccine on April 4, 2021, and the second dose on April 28 same year.

She was admitted to the neurology service of a referral hospital in Peru after three weeks of illness characterized by severe headache, the sensation of thermal rise, conciliation insomnia, and transitory episodes of environment disconnection that began 3 days after receiving the second vaccine dose.

The neurologist evaluated the patient and found that she was confused and unresponsive to simple commands with a tendency to mutism, bilateral pyramidal tract lesions, generalized hyperreflexia, and no signs of meningeal irritation.

Suspecting acute encephalitis, we performed a brain computed tomography (CT) without contrast, finding no pathological results. In addition, we examined the cerebrospinal fluid (CSF) without suggestive findings of infection. We equally prescribed blood count and basic biochemical serum tests that showed no abnormalities. Due to the suspicion of autoimmune encephalitis, we started a five-day dual immunosuppressive treatment scheme with methylprednisolone (1 g daily). We added immunoglobulin (0.4 g/kg daily) on the second day of admission after carrying out serum and CSF tests.

**Fig. 1:**
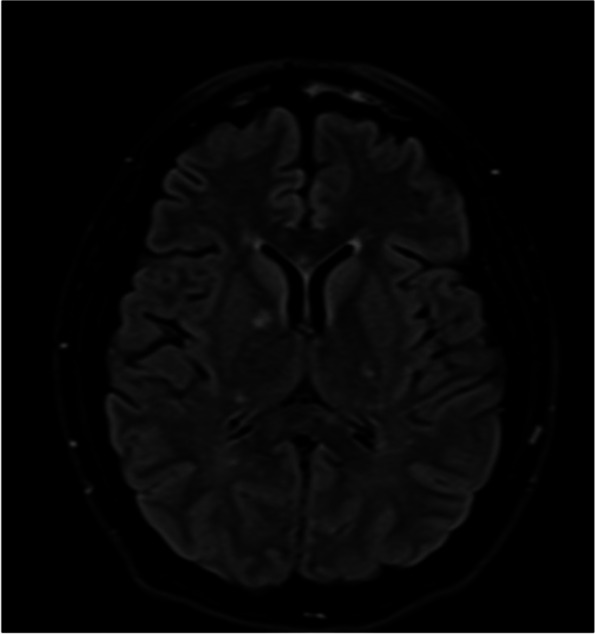
Brain magnetic resonance imaging: T2/FLAIR sequence found small hyperintense nonspecific demyelinating lesions at the periventricular region, internal capsule, and bilateral subcortical areas

On the eighth day after admission, we performed brain magnetic resonance imaging (MRI) with contrast that revealed nonspecific demyelinating lesions at the periventricular region, right cerebellum, internal capsule, and bilateral subcortical areas (Fig. 1). Due to the disconnection with the environment episodes, we performed a prolonged electroencephalogram examination that did not record associated epileptiform activity.

The serum and CSF studies were negative for inflammatory disease (oligoclonal bands in serum and CSF), systemic autoimmune pathologies (C3, C4, antinuclear antibodies, antineutrophil cytoplasmic antibodies, extractable nuclear antigens, rheumatoid factor), neoplastic causes (Pap smear in CSF, flow cytometry and immunofixation in CSF, tumor markers: CEA, CA 125, CA 15–3, AFP, CYFRA 21.1) and associated infectious causes (serological test for syphilis, test for toxoplasmosis, rubella, cytomegalovirus, herpes simplex, viral load of cytomegalovirus, John Cunningham virus in serum and CSF, herpes virus in CSF, HIV, Epstein-Barr virus in serum and CSF, human T-lymphotropic virus type I and II, adenosine deaminase and Koch bacillus in CSF). The cervical, thoracic, abdominal, and pelvic CT scans with contrast excluded the presence of neoplasms.

**Fig. 2:**
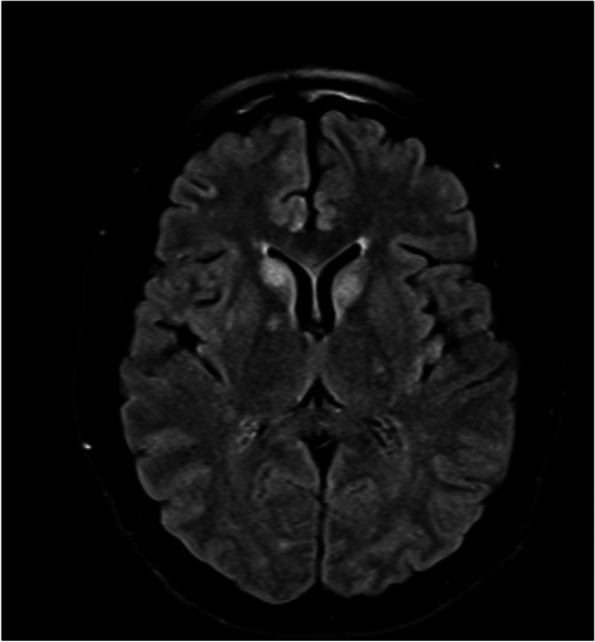
Brain magnetic resonance imaging: T2/FLAIR sequence finding well-defined focal hyperintense lesions at the bilateral caudate nucleus, and demyelinating lesions at the bilateral subcortical areas, already observed in a previous imaging study

During the first three weeks of hospitalization, the patient improved clinically (consciousness, speech emission, and coherency); however, after three weeks of hospitalization and awaiting the autoimmune encephalitis panel results, the patient’s condition worsened, with sustained confusion, apathy-abulic, and gait apraxia. For this reason, we requested another brain MRI that demonstrated new well-defined hyperintense lesions at the bilateral caudate nucleus (Fig. 2).

We started a new cycle of methylprednisolone 1 g daily for five days, with subsequent maintenance therapy of 1 mg/kg of prednisone. Post 1 week of starting the new cycle, the patient improved significantly. The autoimmune encephalitis panel results of the cerebrospinal fluid (NMDA, CASPR2, AMPA 1, AMPA 2, GABA, and LGI 1) and anti-MOG were negative. We then concluded the diagnosis of seronegative autoimmune encephalitis associated with the COVID-19 vaccine. The patient was discharged after a 2-months hospital stay and received outpatient follow-up with oral prednisone without reporting clinical relapses.

## Discussion and conclusion

The annual incidence of encephalitis is estimated at 5–8 cases per 100,000 people, with autoimmune encephalitis being the third most common cause of encephalitis, after infectious and acute disseminated encephalomyelitis (ADEM) [8]. Within the spectrum of autoimmune encephalitis, the subtype with antibodies against the N-methyl-D-aspartate receptor (NMDAR) is the most frequent and represents approximately 1% of all admissions of young adults in intensive care units. However, some cases present without detectable antibodies, are considered seronegative, and require a more specific diagnostic approach [8, 9].

The increase in COVID-19 vaccination coverage has allowed for the monitoring of possible adverse effects, most being neurologically mild. However, cases of encephalitis and ADEM have been reported in some countries with viral vectors [10], mRNA [11], and inactivated virus [12] vaccines. Postvaccination encephalitis is associated with antineural antibodies or seronegative in pathophysiological association with an immune-mediated response triggered by the vaccination process in a person [10–13]. Most of the cases reported were female, developed symptoms after receiving the first dose, and present a median of 9 days postvaccination [13].

The inactive whole virus vaccine prepared by inoculating Verda Reno cells with the WIV04 and HB02 SARS-CoV-2 strain, produced by the Sinopharm laboratory, has been widely used in Asia and Latin America [14]. Our present case is the first case reported in this region to our knowledge.

The clinical presentation of a previous case in Jordan of autoimmune encephalitis due to this vaccine manifested clinically as generalized tonic-clonic seizures with corroborated epileptiform activity on the electroencephalogram [15]. This differs from our case, which was severe, and manifested clinically as an altered state of consciousness without epileptiform activity, postulating a diverse manifestation in these patients without a single pattern.

Cases of nonspecific periventricular lesions have been reported with CoronaVac (manufactured by Sinovac laboratory) vaccination, similar to BBIBP-CorV (Sinopharm). This explains a similar radiological behavior associated with these vaccines [16]. In our patient, multiple nonspecific periventricular demyelinating lesions were observed in the bilateral caudate nucleus, a finding not reported to date in autoimmune encephalitis associated with COVID-19 vaccination [15, 17–22]. Like other cases of immune-mediated encephalitis, she was placed on immunosuppressive treatment with corticosteroids, which usually leads to a favorable response in most cases [8, 10–13]. However, our patient presented an early clinical relapse, prompting a new cycle with corticosteroid therapy, leading to a favorable response and without relapse during outpatient follow-up.

In conclusion, we report the first case of severe encephalitis associated with BBIBP-CorV (Sinopharm) vaccination in Latin America, with atypical imaging findings, an early clinical relapse following treatment, and a favorable response to corticosteroid therapy as an immunosuppressive supplementary treatment.

## Data Availability

All data generated or analyzed during this case report are included in it published version.

## Abbreviations

WHO: World Health Organization
COVID-19: Coronavirus disease
SARS-CoV-2: Severe Acute Respiratory Syndrome Coronavirus 2
CT: Computed tomography
CSF: Cerebrospinal fluid
MRI: Magnetic resonance imaging
CEA: Carcinoembryonic antigen
CA 125: Cancer antigen 125
CA 15–3: Cancer antigen 15–3
AFP: Alpha-fetoprotein
CYFRA 21.1: Cytokeratin 19 fragment antigen
HIV: Human immunodeficiency virus
CASPR2: Contactin-associated protein-like 2
AMPA 1: Alpha-Amino-3-Hydroxy-5-Methyl-4-Isoxazole Propionic Acid 1
AMPA 2: Alpha-Amino-3-Hydroxy-5-Methyl-4-Isoxazole Propionic Acid 2
GABA: Gamma-aminobutyric acid
LGI 1: Leucine rich glioma inactivated 1 protein
Anti-MOG: Anti-myelin oligodendrocyte glycoprotein
ADEM: Acute disseminated encephalomyelitis
NMDAR: N-methyl-D-aspartate receptor
